# The role of Inositol 1,4,5 triphosphate kinase C in the pathogenesis of Kawasaki disease

**DOI:** 10.1186/1546-0096-9-S1-P295

**Published:** 2011-09-14

**Authors:** Vahid Khajoee, Rae SM Yeung

**Affiliations:** 1 Cell Biology Program, Hospital for Sick Children Research Institute, University of Toronto, Toronto, Ontario, Canada; 2 Departments of Pediatrics, Immunology, and Medical Sciences, University of Toronto, Toronto, Ontario, Canada

## Background

Kawasaki disease (KD) is a childhood multisystemic vasculitis resulting in the development of coronary aneurysms. Functional polymorphism in *Inositol 1*,*4*,*5-triphosphate kinase C* (*ITPKC*) has recently been identified and linked to KD susceptibility and severity. ITPKC acts as a negative regulator of T-cell activation through the inhibition of Ca^2+^/Nuclear factor of activated T-cells (NFAT) signalling pathway. *Lactobacillus casei* cell wall extract induced coronary arteritis is an animal model of KD dependent on superantigenic activity.

## Objective

To determine the role of ITPKC in the pathogenesis of KD.

## Methods

To assess the role of ITPKC in lymphocyte activation, mouse splenocytes were stimulated with superantigen and T-cell proliferation, cytokines production, Ca^2+^ flux and ITPKC protein expression were measured by ^3^H thymidine, ELISA, flow cytometry, and Western blot, respectively. To confirm the results in a human system, lymphocyte activation was determined in a human cell line after siRNA knockdown.

## Results

ITPKC was upregulated at both mRNA and protein levels by mouse splenocytes following superantigen stimulation. Cyclosporin A inhibition of Ca^2+^/NFAT signalling abolished T-cell proliferation and cytokine production following superantigen activation. *ITPKC* knockdown in superantigen activated human lymphocyte cell line could not completely inhibit cytokine production or Ca^2+^ flux.

## Conclusion

Although ITPKC and the Ca^2+^/NFAT signalling pathway were activated in lymphocytes following superantigen stimulation, inhibition of ITPKC was not able to alter lymphocyte activation or Ca^2+^ flux pointing to overlapping or compensatory pathways. These findings may account for the conflicting reports on the association between *ITPKC* polymorphisms and KD in different ethnicities.

